# Meet the Section Editor

**DOI:** 10.2174/1570159X2201230921145913

**Published:** 2023-09-21

**Authors:** Constantinos Marios Mikelis

**Affiliations:** 1 Department of Pharmacy, University of Patras, Patras, Greece

Dr. Mikelis is an Associate Professor in the Department of Pharmacy, University of Patras, Greece and an Adjunct Associate Professor in the Department of Pharmaceutical Sciences, School of Pharmacy, Texas Tech University Health Sciences Center (TTUHSC), Amarillo, TX, USA. He also worked at the National Institute of Dental and Craniofacial Research (NIDCR), at the National Institutes of Health (NIH). In 2014, he became Assistant Professor in the School of Pharmacy at TTUHSC, and was promoted to Associate Professor in 2021. He then moved to the University of Patras in 2021, where he is currently working.
